# Genetic Diversity and mRNA Expression of Porcine *MHC* Class I Chain-Related 2 (*SLA-MIC2*) Gene and Development of a High-Resolution Typing Method

**DOI:** 10.1371/journal.pone.0135922

**Published:** 2015-08-25

**Authors:** Hailu Dadi, MinhThong Le, Hunduma Dinka, DinhTruong Nguyen, Hojun Choi, Hyesun Cho, Minkyeung Choi, Jin-Hoi Kim, Jin-Ki Park, Nagasundarapandian Soundrarajan, Chankyu Park

**Affiliations:** 1 Department of Animal Biotechnology, Konkuk University, Seoul, 143–701, Korea; 2 Animal Biotechnology Division, National Institute of Animal Science, Suwon, 441–350, Korea; University of Sydney, AUSTRALIA

## Abstract

The genetic structure and function of MHC class I chain-related (*MIC*) genes in the pig genome have not been well characterized, and show discordance in available data. Therefore, we have experimentally characterized the exon-intron structure and functional copy expression pattern of the pig *MIC* gene, *SLA-MIC2*. We have also studied the genetic diversity of *SLA-MIC2* from seven different breeds using a high-resolution genomic sequence-based typing (GSBT) method. Our results showed that the *SLA-MIC2* gene has a similar molecular organization as the human and cattle orthologs, and is expressed in only a few tissues including the small intestine, lung, and heart. A total of fifteen *SLA-MIC2* alleles were identified from typing 145 animals, ten of which were previously unreported. Our analysis showed that the previously reported and tentatively named SLA-MIC2*05, *07*, and *01* alleles occurred most frequently. The observed heterozygosity varied from 0.26 to 0.73 among breeds. The number of alleles of the *SLA-MIC2* gene in pigs is somewhat lower compared to the number of alleles of the porcine *MHC* class I and II genes; however, the level of heterozygosity was similar. Our results indicate the comprehensiveness of using genomic DNA-based typing for the systemic study of the *SLA-MIC2* gene. The method developed for this study, as well as the detailed information that was obtained, could serve as fundamental tools for understanding the influence of the *SLA-MIC2* gene on porcine immune responses.

## Introduction

The major histocompatibility complex (MHC) is an essential component of the adaptive immune system for all vertebrates. One of the most remarkable characteristics of the *MHC* genes is the presence of extreme polymorphism within loci [1,2]. However, among the genes within the porcine *MHC* class I region, the detailed characteristics and functions of the *MHC* class I chain-related sequences (*MIC*) are not well understood.

The *MIC* gene was first described in primates and other mammals [3]. More than one functional *MIC* gene has been identified in several species; in addition, a number of pseudogenes have been reported [4,5]. Seven *MIC* genes were identified in the human genome, including *MICA* and *B*, which produce functional transcripts, and *MICC*-*G*, which is nonfunctional [6]. Analysis of the cattle genome [7] has led to the identification of three complete *MIC* genes within the *MHC* class I region, temporarily referred to as *BoLA MIC1-3*. In pigs, two *MIC* genes, *SLA-MIC1* and *2*, have been reported [8]; *SLA-MIC2* is functional, whereas *SLA-MIC1* is a truncated pseudogene [5,9–11].

As a member of the MHC class I system, MIC has a similar molecular structure to classical MHC class I molecules. The organization of MIC proteins consists of one transmembrane, and one cytoplasmic, and three external (α1–3) domains, which are encoded by six exons [3,8,12]. Distinguished from their classical MHC class I counterparts, the MIC protein binds neither β_2_-microglobulin (β_2_m) nor present class I peptides [13,14]. In addition, *SLA-MIC2* expression is not affected by interferon, which is the main regulatory factor for classical *MHC* I and II [15]. On the other hand, the MIC protein acts as ligand of NKG2D, a transmembrane receptor, activating the cytolytic response, which is found in many cells within the immune system, including the natural killer cells γδ Τ and αβ CD8+ T [16,17]. In humans, *MIC* is transcribed in several immune cells and most epithelial tissues. However, cell surface expressions of *MIC* were reported only from freshly isolated endothelial cells, fibroblasts [18], and gastric epithelium [13]. On the other hand, there were reports showing the up-regulation of transcripts and cell surface protein expression of MIC in many cell lines, including immune cells when stimulated with cellular stress inducers [14,19]. Consistently, heat-shocked, viral-infected, and cellular-transformed upregulation of *MIC* has led to the impression that it is probably a ‘marker of stress’, especially in epithelial cells [13,20,21].

Several studies have demonstrated the possible associations between *MIC* genes and diseases [22–24]. For example, a strong association has been shown between specific *MICA* alleles and autoimmune disorders such as Behˎçet’s disease [24,25]. Other studies have also demonstrated an association between *MICA* alleles and human brucellosis resistance or susceptibility [22–24]. However, the linkage disequilibrium to classical *MHC I* or other genes in the *MHC* region may complicate disease association studies [26]. Therefore, high-resolution typing of candidate genes may be beneficial to the reduction of possible bias.

In this study, we experimentally confirmed the molecular organization and expression pattern of *SLA-MIC* transcripts, characterized the polymorphism using a genomic DNA-based high resolution typing method, and performed a comparative analysis of *MIC* genes for seven mammalian species. Our results contribute to a more complete understanding of the molecular complexity and genetic variation of *SLA-MIC2* and provide novel tools for genotyping.

## Materials and Methods

### Animals and preparation of DNA

The Institutional Animal Care and Use Committee (IACUC) of Konkuk University approved the ear tissue and peripheral blood sampling methods. The IACUC approval number of this study is KU13101.

Initially, 28 samples were selected on the basis of *SLA1* genotypes (22 different alleles, data not shown) as reference samples for the development of the *SLA-MIC2* typing method. To estimate *SLA-MIC2* diversity, we further typed 117 randomly selected pigs from seven different pig breeds and resulted in typing a total of 145 animals: 22 Seoul National University (SNU) miniature pigs, 25 Korean native pigs (KNPs), 13 National Institutes of Health (NIH) miniature pigs, 22 Duroc pigs, 20 Landrace pigs, 19 Yorkshire pigs, and 24 Berkshire pigs. Genomic DNA was extracted from 0.5 g of ear tissue obtained by ear punching, or 1 mL peripheral blood containing 6% ethylene diamine tetra acetic acid (EDTA), according to a previously described protocol [27].

### Polymerase chain reaction (PCR) primer design

We aligned available genomic sequences of *SLA-MIC2* (accession numbers CT737281, AJ251914, and NM_001114274) using ClustalW software (http://www.genome.jp/tools/clustalw/), and analyzed the exon-intron organization. After we determined the correct exon-intron sequences, primers for the amplification of *SLA-MIC2* genomic DNA (gDNA) and complementary DNA (cDNA) were designed against a reference sequence (CT737281) using Primer Designer software (Version 2.0; Scientific and Educational Software, State Line, PA, USA). Primer sequences, annealing temperatures, and the size of the PCR products are summarized in Table 1.

**Table 1:** Primer sequences and amplification conditions used for *MIC2* analysis.

Target regions	Primer ID	Primer sequences (5’-3’)	Annealing temperature (°C)	Product size (bp)
		**gDNA-PCR**		
*MIC-2* (Exons 2 to 4)	MIC2-gDNA-F1	TGTCCTCTGCTTGCCGATCTC	66	2512
	MIC2-gDNA-R1	ATCCAGAACCACCTAGATCC		
		**Sequencing primers**		
*MIC2*: Exon 2	MIC2-E2sF	TTCTGGCCCCTTGTACACAT	55	
	MIC2-E2sR	TCCATGCTCAGCTCACAGAC		
*MIC2*: Exon 3	MIC2-E3sF	CCTTGACTCAGCAGCACAGG	55	
	MIC2-E3sR	GGACTGACCAGAAGAGCAAG		
*MIC2*: Exon 4	MIC2-E4sF	TGCATGAAGGCTCAGCCAG	55	
	MIC2-E4sR	AGCCTGGCCTCTGGATCTC		
*MIC2*-cDNA		**cDNA PCR 5’ UTR to 3’ UTR**		
	MIC2-cDNA-F	GAGCGAGTGTCCCATTTGGGA	48	1262
	MIC2-cDNA-R	GGCCAGAACAGGGAGTTGAATTC		
		**cDNA PCR partial, exon 2 to 3’ UTR**		
	MIC2-cDNA-F1	GGTACAACTTCACGGTGATG	48	1080
	MIC2-cDNA-R	GGCCAGAACAGGGAGTTGAATTC		
		**cDNA PCR partial, exon 3 to 3’ UTR**		
	MIC2-cDNA-sF*	GGAGAAGACGTGCGACATGG	48	670
	Oligo dT(17)	TTTTTTTTTTTTTTTTT		
		**Full-length cDNA sequencing primers**		
MIC2-cDNA	MIC2-cDNA-sF	GGAGAAGACGTGCGACATGG	55	
	MIC2-cDNA-sR	CTCTGTGAAGCTGGTCCAGG		
		**GAPDH PCR primers**		
*GAPDH*	GAPDH-F	ACTCACGGCAAATTCAACGGC	48	294
	GAPDH-R	ATCACAAACATGGGGGCATCG		

*MIC2-cDNA-sF primer was used for both PCR and cDNA sequencing.

### Amplification of *SLA-MIC2* and direct sequencing

PCR reactions were performed in a 20 μL volume containing 50 ng DNA, 0.5 μM of each primer, 200 μM dNTPs, PCR buffer [10 mM Tris (pH = 8.3), 50 mM KCl, and 1.5 mM MgCl_2_], and 0.5 U LA-Taq polymerase (Takara Biotechnology Inc., Otsu, Shiga, Japan). PCR thermal cycling was performed using a T-3000 thermal cycler (Biometra, Goettingen, Germany), and consisted of an initial denaturation of 95°C for 5 minutes, followed by 35 cycles of 35-second denaturation at 94°C, 45-second annealing at 66°C, and 2-minute extension at 72°C; a final extension at 72°C for 10 min was then performed. PCR products were confirmed by electrophoretic separation on a 1.5% agarose gel in 1X Tris-acetate-EDTA (TAE) buffer for approximately 25 minutes at 100 V. The gel was stained with ethidium bromide and visualized under ultraviolet light.

For the direct sequencing of PCR products, 5 μL of the product was incubated with 4 U exonuclease I (Fermentas, St. Leon-Rot, Germany) and 0.8 U shrimp alkaline phosphatase (USB Corporation, Cleveland, OH, USA) for 30 min at 37°C in 2.5× reaction buffer to degrade primers and dephosphorylate dNTPs that were not consumed in the amplification reaction. The purification reaction was stopped by a 15-minute incubation at 80°C. Sequencing reactions were performed using the ABI PRISM BigDye Terminator Cycle Sequencing Kit (Applied Biosystems, Foster City, CA, USA) using specific forward and reverse primers for each exon according to the manufacturer’s instructions. The products were analyzed using an automated DNA analyzer (Applied Biosystems, Foster City, CA, USA). All sequences were checked for ambiguous bases and manually edited using BioEdit V7.0 software [28].

### Confirmation of new *SLA-MIC2* alleles

New *SLA-MIC2* alleles or any alleles that appeared for the first time in our analysis were confirmed by cloning PCR products and bidirectional sequencing. PCR products were gel purified using a QIAquick gel extraction kit (QIAGEN, Venlo, the Netherlands) and ligated into the pGEM-T Easy Vector System (Promega Corporation, Madison, WI, USA). Ligation products were transformed into *Escherichia coli* DH-5α competent cells. These cells were grown overnight on agar containing 50 μg/mL ampicillin, 40 mg/mL X-gal (Norgen Biotek Corp. Thorold, ON, Canada), and 100 mM Isopropyl β-D-1-thiogalactopyranoside (IPTG) (Thermo Scientific, Waltham, MA, USA) at 37°C. Five white colonies were picked from each ligation to amplify inserts using T7 and SP6 universal primers. Sequencing reactions were performed using exon-specific primers for each exon, as indicated in Table 1. After confirming the accuracy of the sequencing results by manual inspection of the chromatograms, a sequence similarity search (BLAST) against the NCBI (National Center for Biotechnology Information) database, as well as local *SLA-MIC2* databases, was performed. The complete sequences of *SLA-MIC2* exons 2, 3, and 4 were aligned using CLUSTALW [29], as implemented in MEGA 6.0 [30].

### RNA isolation, reverse transcription PCR (RT-PCR), and *SLA-MIC2* cDNA typing

Total RNA was isolated from fifteen different tissues (liver, stomach, lung, small intestine, heart, skin, tongue, spleen, muscle, large intestine, testis, kidney, ovary, neocortex, and olfactory epithelium) of a nine-week-old female pig using the R&A-BLUE total RNA extraction kit (iNtRON Biotechnology, Seoul, Korea), following the manufacturer’s protocol. Reverse transcription was carried out in a 25 μL reaction using oligo-(dT)_17_ and Superscript III reverse transcriptase (Invitrogen, Carlsbad, CA, USA) for 50 min at 50°C, and incubated for 15 min at 72°C to stop the reaction. RT-PCR was performed using a T-3000 thermal cycler. The PCR mixture consisted of 50 ng cDNA, 0.5 U Super-Therm DNA polymerase (JMR Holdings, Kent, UK), 0.5 μM primers (Table 1), 1X PCR reaction buffer (1.5 mM MgCl_2_), and 0.1 mM dNTPs. The amplifications were performed in a 20 μL reaction volume. The thermal cycling profile included a 5-minute denaturation step at 95°C, followed by 35 cycles of denaturation for 1 minute at 95°C, annealing for 1 minute at 48°C, and extension for 1 minute at 72°C, followed by a final extension of 5 minutes at 72°C. Direct sequencing was performed on the products using cDNA sequencing primers (Table 1).

### Analysis of *SLA-MIC2* expression by semi-quantitative RT-PCR

To evaluate the levels of *SLA-MIC2* mRNA in tissues, primers for amplifying full-length (5′ to 3′ UTRs) and partial sequences (exon 2 to 3′ UTR) of *SLA-MIC2* cDNA were designed, and semi-quantitative RT-PCR was performed. PCR mixtures and RT-PCR conditions were identical to the experimental conditions described above, except that the number of cycles was decreased to 32. Glyceraldehyde-3-phosphate dehydrogenase (*GAPDH*) was used as a control for experimental variation. The photodensity ratio was calculated by comparing the density of the *SLA-MIC2* amplicon relative to that of *GAPDH* using Image Studio Analysis Software Version 4.0 (LI-COR Biosciences, USA).

### Statistical and phylogenetic analysis

Population statistics, including allele frequencies, number and effective number of alleles, observed and expected heterozygosity, and the Hardy-Weinberg equilibrium, were estimated by using POPGENE 1.32 [31]. Phylogenetic analysis of *SLA-MIC2* alleles was performed using the neighbor-joining method [32] with bootstrap analysis of 1000 replicates, and evolutionary distances among alleles were calculated using the Kimura 2-parameter model [33] using MEGA 6 software [30].

## Results and Discussion

### Characterization of *SLA-MIC2* gene structure

**Fig 1:**
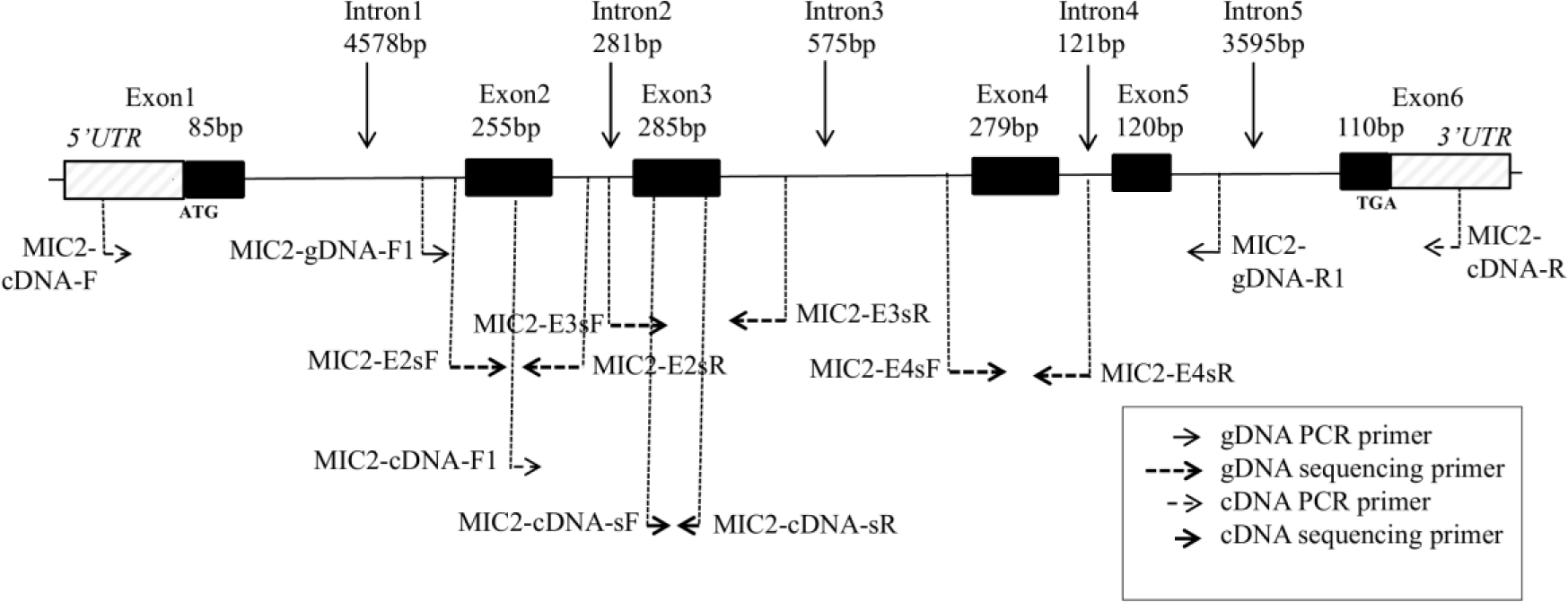
General strategy of genomic sequence-based genotyping for pig *SLA-MIC2*. The diagram shows the location of each primer for PCR and sequencing. The sizes (bp) of introns and exons are indicated.

**Fig 2:**
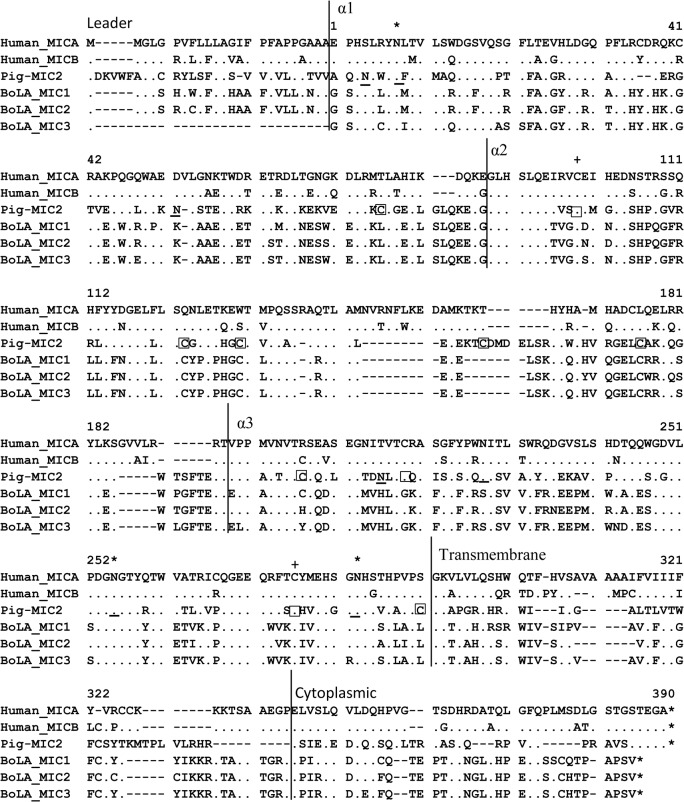
Comparison of amino acid sequences of MIC genes among pigs, humans, and cattle. A representative sequence of each functional *MIC* gene from each species was selected, and amino acid sequences were compared throughout the entire coding region to evaluate sequence conservation. The accession numbers for the sequences are *BoLA-MIC1* (BK006541), *BoLA-MIC2* (BK006542), and *BoLA-MIC3* (BK006543) for cattle, and *MICA* (NM_000247) and *MICB*- (NM_005931) for humans. Potential sites for N-linked glycosylation are underlined, and cysteine residues are indicated in squares for *SLA-MIC2*. Gaps are indicated by dashes and identical residues are indicated by dots. Stars above the sequences indicate conserved N-linked glycosylation sites, and plus signs above the sequences indicate a cysteine residue that is conserved across species. The starting points of protein domains are indicated above the annotated sequence, and the numbers above the sequence indicate the number of amino acids starting from the α1 domain excluding the leader peptide.

We designed sets of PCR primers against the *SLA-MIC2* sequence contained within a bacterial artificial chromosome (BAC) sequence, AJ251914, and attempted to amplify the region between introns 1 and 4, or the full-length *SLA-MIC2* cDNA, according to the exon-intron information of the pig *SLA-MIC2* gene from a previous report [5]. Following the results of *MICA* and *MICB* expression from humans [34], we selected the lung and small intestine as initial RNA sources [13,21,34]. However, we were unable to obtain amplicons from either genomic DNA PCR or RT-PCR. Through additional database searches, we identified another BAC sequence (CT737281) that contained the *SLA-MIC2* gene, but with a sequence discrepancy from the end of exon 4 to exon 6 of *SLA-MIC2*, as compared to AJ251914. We also realized that the exon-intron organization from AJ251914 was different from the current *in silico* annotation (NM_001114274) of the *SLA-MIC2* gene according to the Ensemble genome browser (http://asia.ensembl.org/index.html). To resolve the discrepancy, we performed RT-PCR with *SLA-MIC2*-specific forward primers and a poly A specific oligo (dT)_17_ reverse primer. Among the several primer combinations used, a *SLA-MIC2* exon 3-specific forward primer (MIC2-cDNA-sF) produced a 670-bp cDNA product. From this result, we were able to identify the differences in both the nucleotide sequence and the position of exon-intron boundaries for *SLA-MIC2* exons 5 and 6 between the reported information and our findings, resulting in the precise characterization of the exon-intron structures of full-length *SLA-MIC2* cDNA (Fig 1 and S1 Fig). The porcine *MIC2* gene encodes a polypeptide of 374 amino acids consisting of a leader sequence (exon 1), three extracellular domains α1–3 (exons 2, 3 and 4), a transmembrane domain (exon 5), and a cytoplasmic domain (exon 6) (Fig 2 and S1 Fig), which were defined by comparative analysis with human *MICA*.

### Analysis of *SLA-MIC2* expression in 15 different pig tissues

**Fig 3:**
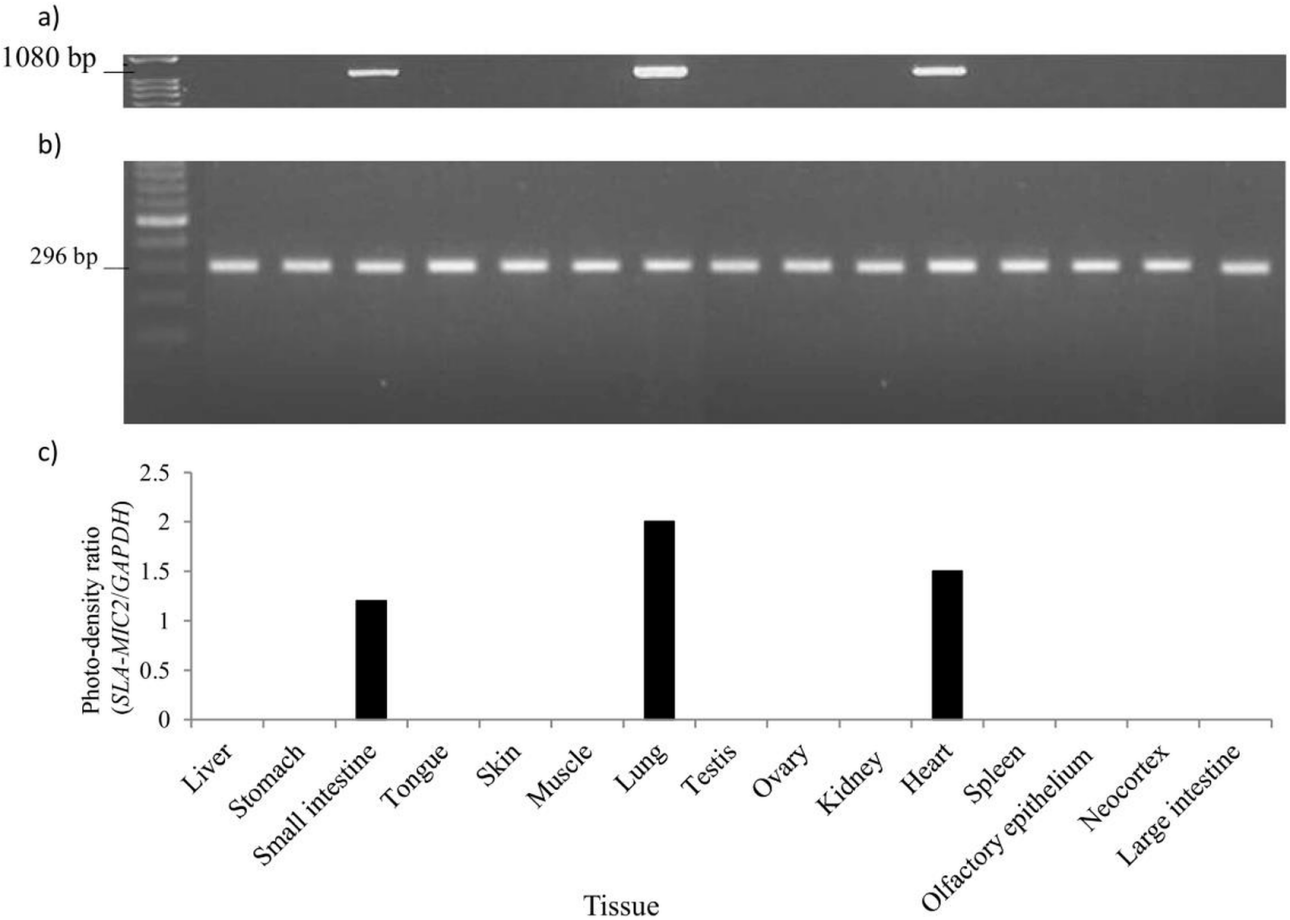
Comparison of the mRNA expression levels of *SLA-MIC2* in various pig tissues. **a)** The RT-PCR products (1080 bp) of *SLA-MIC2* exons 2 to 6 in different tissues, amplified from RNA isolated from nine-week-old male pigs. **b)** Standard *GAPDH* gene expression levels in different tissues, as visualized by the intensity of RT-PCR product staining on an agarose gel. **c)** The photodensity ratios between the amplified *SLA-MIC2* and *GAPDH* products.

We examined the expression of *SLA-MIC2* in 15 different pig tissues using semi-quantitative RT-PCR. The comparison of band intensity and semi-quantitative measures of *SLA-MIC2* expression (photodensity ratio) showed that *SLA-MIC2* was expressed in only the small intestine, lung, and heart, with the most abundant expression in the lung (Fig 3). These findings suggested that the expression patterns of *MIC* genes among different species are not identical, and that the expression of *MIC* genes could vary even within a species. The difference in the expression pattern between pigs and humans suggests the presence of possible differences between the *MIC*-related immune systems of the two species. Further studies are necessary to understand the consequences of these differences.

### Development of a genomic DNA-based high-resolution typing method for *SLA-MIC2*

The availability of an efficient typing method for highly polymorphic loci is fundamental to understanding the underlying biology associated with their genetic polymorphisms, especially for MHC or related molecules. In contrast to a considerable amount of data from human *MIC* gene [3,13,22–24,35,36] studies, detailed information on the genetic diversity of *SLA-MIC2* has not been available. Therefore, we carried out the development of a high-resolution typing method of the *SLA-MIC2* gene covering exons 2, 3, and 4, which is the region of functional importance for *MICA* and *B*, required for the typing of *MICs* in humans.

After we confirmed the gene structure of *SLA-MIC2* (S1 Fig), systematic trials of different primer sets to amplify all of the *SLA-MIC2* alleles regardless of the existing sequence variations were performed. PCR amplification, cloning of PCR products, and subsequent sequence analyses were performed using genomic DNA from 28 animals that were selected based on the diversity of *SLA-1* corresponding to 22 different *SLA-1* alleles (data not shown). The possible linkage between *SLA1* and *SLA-MIC2*, which are ~0.7 Mb apart [11], should allow maximum diversity of the *SLA-MIC2* gene by choosing animals with different *SLA-1* alleles.

Among the pairs of primers tested, MIC2-gDNA-F1 and MIC2-gDNA-R1 (Table 1) showed successful amplification of *SLA-MIC2* for all samples (S2 Fig). The general strategy of our *SLA-MIC2* high-resolution typing method is described in Fig 1. PCR amplification using the gDNA PCR primer set resulted in a 2512-bp PCR fragment, covering partial intron 1, exon 2, intron 2, exon 3, intron 3, exon 4, intron 4, exon 5, and partial intron 5.

For sequence-based discrimination of *SLA-MIC2* alleles, several sequencing primers were designed for each of exons 2–4. Among them, MIC2-E2sF and MIC2-E2sR for exon 2, MIC2-E3sF and MIC2-E3sR for exon 3, and MIC2-E4sF and MIC2-E4sR for exon 4 showed consistent results when used for direct sequencing (Table 1), leading to the establishment of the sequence analysis method. To assemble the sequence information of each exon into a single sequence contig, the eight possible combinations of assembled typing results were compared to the available sequence information of existing alleles in the public database, or to previously confirmed alleles in our local *SLA-MIC2* database. Complete matches to the known alleles in public or local databases were determined to be valid alleles. In cases of heterozygotes, alleles were separated according to the complete sequence identity to the existing alleles in our *SLA-MIC2* database. However, due to the possible appearance of unknown alleles, we originally carried out the cloning, sequencing, and subsequent allelic characterization in all genotyped samples to increase the amount of information in our *SLA-MIC2* allele database. Since the probability of a hybrid exon occurring between different alleles in the population by genetic recombination should be extremely low, we believed that the best-fit prediction approach for allele interpretation should result in accurate typing results. Because we were able to amplify the target region in all samples using the initial set of primers, we did not analyze the sequence variations of intronic regions of different *SLA-MIC2* alleles, which was necessary for the development of typing methods for *MHC* class II genes in pigs [37].

### Verification of *SLA-MIC2* typing accuracy

The reliability of our *SLA-MIC2* genomic sequence-based typing (GSBT) method was evaluated by cloning PCR products and subsequent sequencing, followed by comparison to the typing results for cDNA and artificial heterozygote samples. First, the typing results for all of the newly identified *SLA-MIC2* alleles, or genotyping results that appeared for the first time in our analysis, were subjected to cloning-based analysis with at least five clones in both the forward and reverse direction. Samples with typing results that contained even a single base mismatch to known alleles were subjected to cloning-based analysis to eliminate any possible typing errors. This process strictly followed the requirements of the SLA Nomenclature Committee of the International Society for Animal Genetics (ISAG) for acceptance of new alleles. At least two PCR reactions were performed for one sample: one for direct sequencing and the other for cloning; the result was a collation of the two reactions. The *SLA-MIC2* GSBT and cloning-based typing resulted in identical outcomes for all comparisons (n = 15 alleles). Second, we performed cDNA typing using the primer set MIC2-cDNA-F and MIC2-cDNA-R for samples with available RNA, and compared the results to those obtained using the *SLA-MIC2* GSBT method. The results were identical for all samples (n = 10). The alleles verified by cDNA typing are shown in Table 2. Third, allelic dropout or preferential amplification is one of the most common genotyping errors, leading to the preferential amplification of a single allele from heterozygotes. The occurrence of allelic dropout was examined by mixing equal concentrations of DNA (50 ng/μL) from five different *SLA-MIC2* homozygote samples containing the alleles MIC2*01, *05, *04, *07, and *kn15. This mixture was used to prepare six different simulated heterozygotes, including SLA-MIC2*01/05, 04/05, 05/07, 01/kn15, 04/kn15, 05/kn15, which were not available from the animals in the study. We found that the genotyping results from the artificial heterozygotes were identical to the predicted genotypes for all samples (data not shown). Combined, these results suggest that the amplification of each allele from at least the confirmed combinations in this study did not appear to be affected by allelic biases in PCR amplification, indicating that our genotyping primers are located in intronic regions of high sequence conservation across breeds of pigs.

**Table 2:** Comparison of the allele frequency of porcine *MIC2* among seven pig breeds using high-resolution genomic sequence-based typing.

Allele	BER	KNP	NIH	SNU	YOR	DUR	LAN	All (n = 145)
MIC2*01^a^	0.208		0.308	0.109	0.167	0.046	0.075	0.117
MIC2*03^a^	0.021	0.120						0.024
MIC2*04^a^		0.280		0.022	0.417			0.103
MIC2*05^a^	0.042	0.020	0.692	0.869	0.056	0.546	0.025	0.300
MIC2*07^a^	0.479				0.139	0.227		0.131
MIC2*kn08		0.100				0.023		0.021
MIC2*kn09					0.056		0.050	0.014
MIC2*kn10	0.125	0.280			0.083		0.100	0.097
MIC2*kn11		0.040						0.007
MIC2*kn12	0.021							0.003
MIC2*kn13						0.023	0.275	0.041
MIC2*kn14							0.050	0.007
MIC2*kn15^a^	0.021					0.114	0.350	0.069
MIC2*kn16^a^	0.083				0.083		0.050	0.031
MIC2*kn17		0.160				0.046	0.025	0.038

Note: SNU, Seoul National University miniature pigs; KNP, Korean native pig; NIH, National institute of Health miniature pig; DUR, Duroc; LAN, Landrace; YOR, Yorkshire; BER, Berkshire.

“^a^” Alleles were verified by cDNA typing. SLA-MIC2*01, *03, *04, *05 and *07 are existing alleles in the GenBank database. Ten new alleles were submitted to GenBank under the accession numbers KM514686, KM514687, KM514688, KM514689, KM514690, KM514691, KM514692, KM514693, KM514694 and KM514695, which were provisionally named as SLA-MIC2*kn08, *kn09, *kn10, *kn11, *kn12, *kn13, *kn14, *kn15, *kn16 and *kn17, respectively. The assignment of tentative names for new *SLA-MIC2* alleles followed SLA Nomenclature Committee guidelines.

Combining genomic PCR and subsequent direct sequencing of PCR products can serve as a reliable technique for the detection of genetic polymorphisms in population studies, due to the presence of a larger number of correct template copies, compared to a smaller number of mutated templates generated during PCR and cloning processes, ultimately leading to more accurate results. This approach was proven successful for the typing of *MHC* class II loci [38]. Considering the high fidelity of typing results from genomic PCR and subsequent direct sequencing analyses, we propose that it may not be necessary to perform bidirectional sequencing when the typing results perfectly match previously confirmed alleles.

### Structural comparison of *MIC* genes among pigs, humans, and cattle

*MICA* in humans encodes a full-length polypeptide of 383 amino acid residues, with the relative molecular mass of 43 kDa [3]; *BoLA MIC1* and *MIC2* consist of 384 amino acids [7]. In contrast, pig *SLA-MIC2* encodes a 374-amino acid polypeptide (Fig 2). When we compared the exon-intron organization of the *SLA-MIC2* gene to that of the reported functional *MIC* genes of cattle and humans, the organization was almost identical except for slight differences in the length of certain exons (Fig 2).

**Fig 4:**
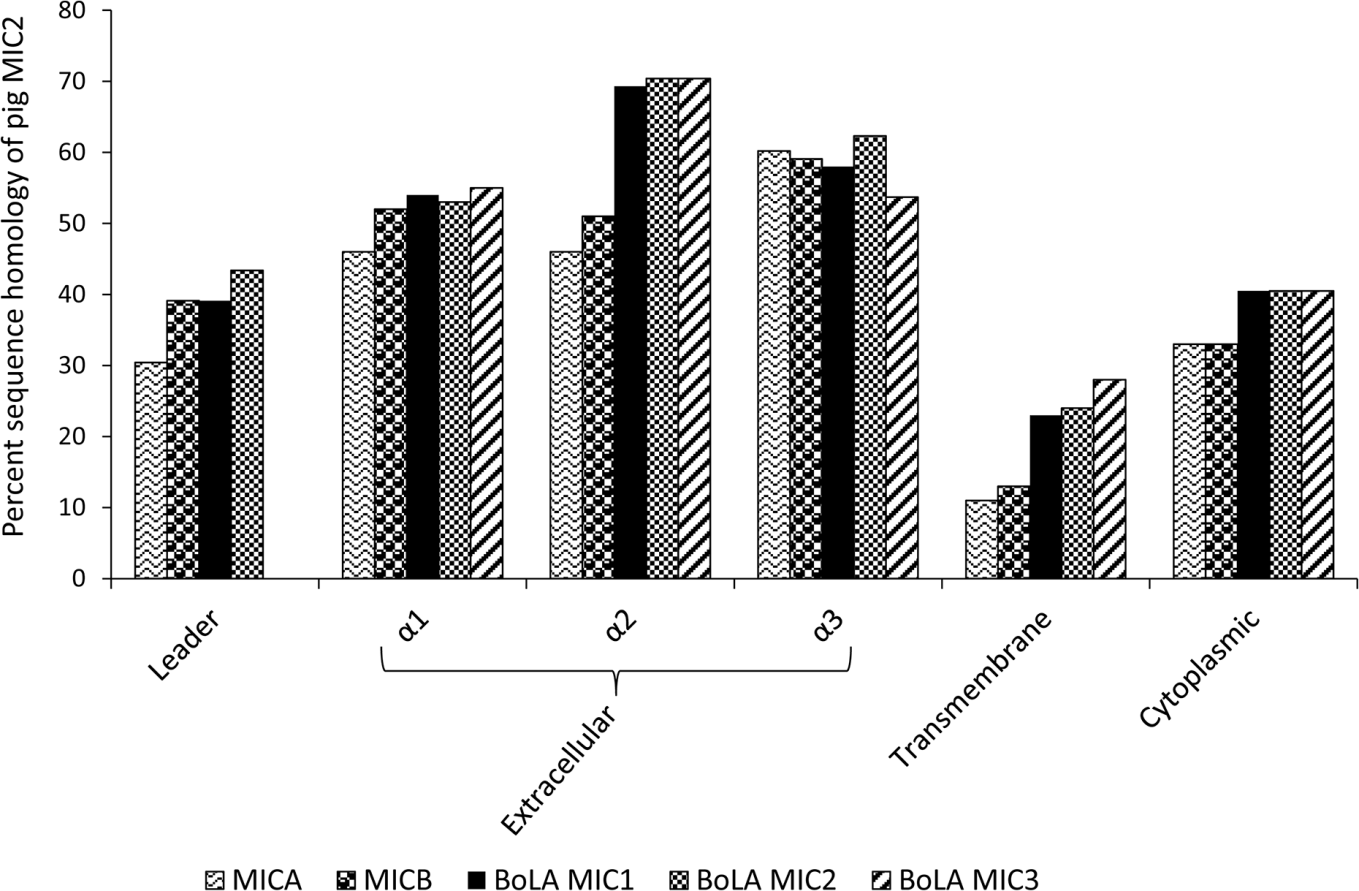
Amino acid sequence similarities between swine leukocyte antigen *SLA*-*MIC2* and *MIC* orthologs of humans and cattle. The different protein domains are indicated below the x-axis. *MICA* and *MICB* are from humans; *BoLA-MIC1*, *2*, and *3* are from cattle.

The similarities between the amino acid sequence of *SLA-MIC2* and the orthologs in cattle and humans are shown in Fig 4. Depending on the domain, *SLA-MIC2* shared approximately 11–70% amino acid sequence similarity with these orthologs. When we compared *MIC* sequences between humans and cattle, *SLA-MIC2* extracellular domains α1 and α2 showed 2 and 6 amino acid inserts or deletions (indel), respectively, while no length variation was observed for the α3 domain (Fig 2). The transmembrane and cytoplasmic domains displayed less sequence similarity than extracellular domains with 14 and 8 indel, respectively. For the leader peptide region, the sequence similarity was weak among the different species. The high sequence similarity of the extracellular domains α1–3 suggests that structural conservation of the extracellular domains is important for protein function. The amino acid sequence identity between pigs and cattle was higher than that between pigs and humans.

Glycosylation is important for protein stability and biological function [39–41]. The effects of glycosylation often depend on the position and number of N-linked oligosaccharides added to a protein chain. We identified seven putative N-linked glycosylation sites (3 sites in α1 and 4 sites in α3) from *SLA-MIC2*, indicating that all the predicted glycosylated residues reside in the extracellular domains (Fig 2). It has been reported that cattle *MICs* have six potential sites for N-linked glycosylation [7], while human *MICA* has eight [3]. Comparing the patterns of amino acid conservation of *MIC* genes in pigs, cattle, and humans, we found three conserved N-linked glycosylation sites (Asn-8, -255, and -283) among the species.

Cysteine is one of the least abundant amino acids in an organism. It is often present in functionally important protein sites. We have detected 10 cysteine residues concentrated in the extracellular domains of *SLA-MIC2*, one in α1 (Cys-76), five in α2 (Cys-99, -123, -130, -158, and -176) and four in α3 (Cys-207, -219, -276, and -291; Fig 2). Human *MICA* contains seven cysteine residues that are also located in the extracellular domains [3]. Pig, cattle, and human genes were shown to share conserved cysteine in the α2 (Cys-99) and α3 (Cys-276) domains (Fig 2). In addition, pigs and cattle were found to share three conserved cysteines in the α2 domain. The differences in positions and the number of cysteines in *MIC* proteins from different species may result in changes in the protein structure and stability [42]. Comparisons of the *MIC* protein structures, which are currently unavailable, are necessary to verify the influence of these variations on the structure and function of *MIC*s in different species.

### Genetic diversity of *SLA-MIC2*

Seven different sequences corresponding to pig *SLA-MIC2* exons 2–4 are currently identifiable using the NCBI database. In this study, we identified 15 different *SLA-MIC2* alleles (Table 2) from typing 145 animals from seven pig breeds which are consisted of 22 non-randomly selected reference individuals (19.3%) of high *SLA-1* allele diversity and 117 randomly selected individuals from seven pig breeds. Therefore, the allele frequencies of *SLA-MIC2* in Table 2 were not estimated entirely from randomly chosen animals for each breed. However, they still should not significantly deviate from the patterns of allele distribution specific to each breed. At the amino acid level, nine *SLA-MIC2* alleles can be discriminated. There are considerably fewer *SLA-MIC2* alleles compared to the number of alleles from *SLA* class I (116 alleles) and class II (167 alleles). This is similar to what is observed in humans, where there are 100 *MICA* alleles in contrast to 3,105 human leukocyte antigen (*HLA*) class II alleles or 9,308 *HLA* class I alleles (Immuno Polymorphism Database [IPD], https://www.ebi.ac.uk/ipd/).

Among the alleles, five were previously reported with tentative names, including MIC2*01, MIC2*05, MIC2*03, MIC*04, and MIC2*07; ten were new alleles. The common alleles, MIC2*05, MIC2*07, and MIC2*01, accounted for approximately 30.3%, 13.1%, and 12% of the *SLA-MIC2* gene pool, respectively. A low frequency was observed for the remainder of the alleles, and the frequency varied among different breeds. The frequency of MIC2*05 was the highest in the NIH and SNU miniature pigs, as well as the Duroc breed. Besides, the three alleles including MIC2-*kn11, MIC2-*kn12, and MIC2-*kn14 were unique to KNPs, Berkshire and Landrace breeds, respectively. Of the new alleles, MIC2*kn12 and MIC2*kn11 were observed from only one and two heterozygote individuals, and MIC2*kn09 and MIC2*kn16 occurred in several heterozygote animals. The remaining new alleles occurred in at least one homozygote and several heterozygote individuals.

We compared 15 *SLA-MIC2* alleles that were identified in this study with the *SLA-MIC2* sequence from BAC CT737281 (S3 Fig). All of the allelic variations that were detected were nucleotide substitutions. We examined the patterns of amino acid changes for each nucleotide substitution in *SLA-MIC2* sequences. Seven non-synonymous and five synonymous coding mutations were detected (S3 Fig). Three polymorphic positions were identified in *SLA-MIC2* exon 2, resulting in three distinguishable *SLA-MIC2* exon 2 sequences. For exon 3, five polymorphic positions were observed, resulting in eight distinguishable *SLA-MIC2* exon 3 sequences. For exon 4, four polymorphic sites defined seven different sequences in exon 4.

Heterozygosity may contribute to increased resistance to infectious diseases. In particular, high heterozygosity at the *MHC* locus has been shown to be beneficial to species by conferring a selective advantage through enhancing resistance to infectious diseases [43]. The average level of observed heterozygosity from 145 typed animals in seven different breeds was 52.1%, ranging from 26% for inbred SNU miniature pigs, to 72.8% for outbred Duroc animals. The average level of expected heterozygosity for the same data set was 63.4%, ranging from 23.6% in SNU miniature pigs to 79.7% in Landrace pigs (Table 3). The levels of *MIC2* heterozygosity among different breeds were diverse and even higher than those of the *SLA* class II genes that were reported in previous studies (0.1–0.69 in *SLA-DQB1* and 0.28–0.77 in *SLA-DRB1*) [37,38]. A potential explanation for this finding is that some animals (19.3%) employed for S*LA-MIC2* typing in this study had non-random distribution of *SLA1* alleles according to our typing strategy. Inbred pigs, including NIH and SNU miniature breeds, showed lower heterozygosity than outbred breeds, as expected. There was no excess or deficiency of heterozygotes in six breeds, which is consistent with previous reports on *MHC* genes [37,44]. Only the Landrace breed (P < 0.001) deviated from Hardy-Weinberg equilibrium in our study, which may be attributed to the limited sample size or use of animals with selected haplotypes according to *SLA-1*.

**Table 3:** Differences in porcine *MIC2* heterozygosity among seven breeds of pigs.

Breed	N	Number of alleles	ne	Het-O	Het-E	HWE (P values)
BER	24	8	3.348	0.625	0.716	0.52
KNP	25	7	4.562	0.56	0.796	0.06
NIH	13	2	1.742	0.307	0.443	0.244
SNU	22	3	1.301	0.26	0.236	0.935
YOR	19	7	4.153	0.666	0.781	0.552
DUR	22	7	2.916	0.728	0.672	0.288
LAN	20	9	4.494	0.5	0.797	0.001**
Total	145	15	6.646	0.521^a^	0.634^b^	

Note: Het-O: observed heterozygosity; Het-E: Nei’s expected heterozygosity; ne: effective number of alleles; HWE shows P-value for heterozygous protein

S deficiency from the Hardy–Weinberg equilibrium likelihood ratio test

**P < 0.00

^a^average observed heterozygosity

^b^average expected heterozygosity

### Phylogenetic analysis

**Fig 5:**
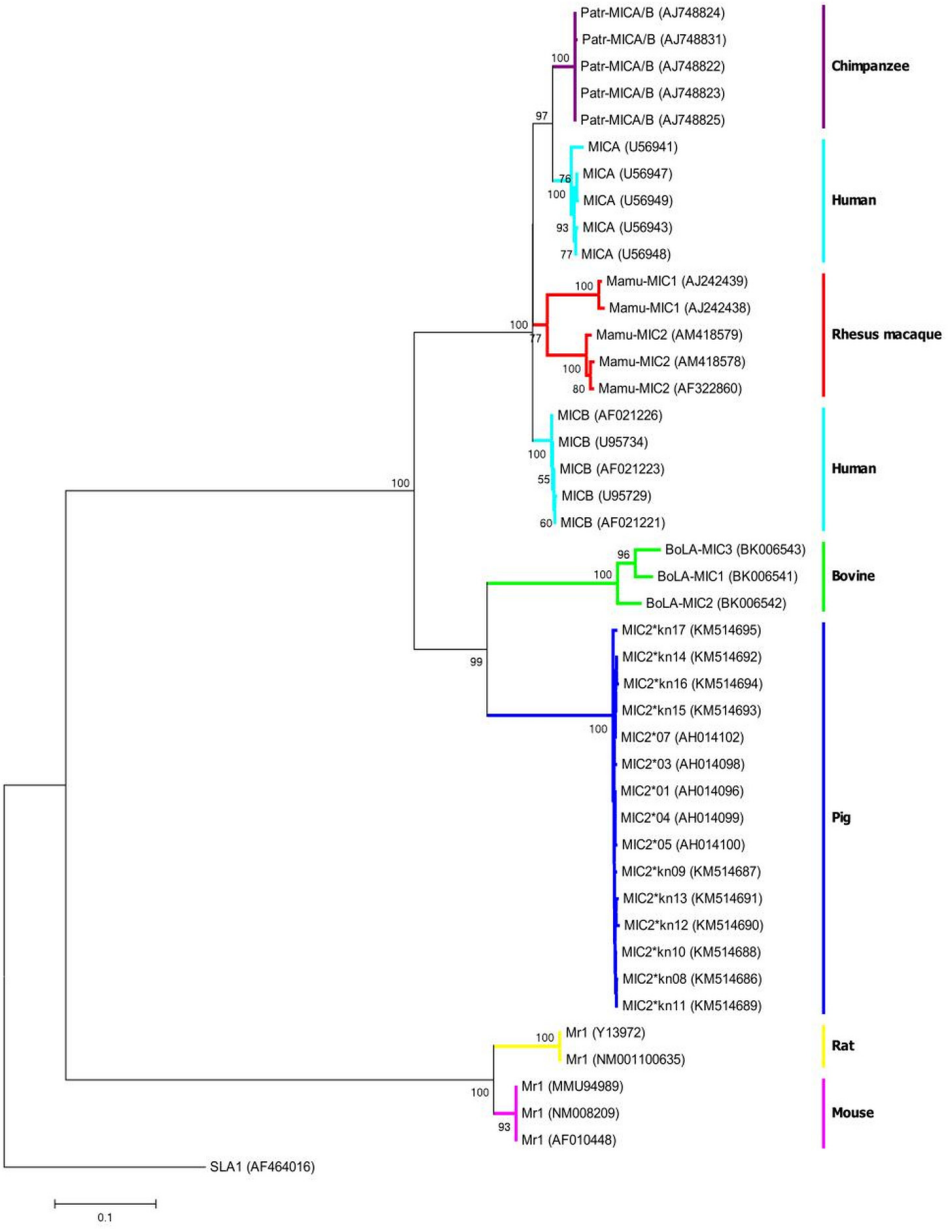
A phylogenetic tree showing the relationships of *MIC* orthologous genes in different mammals including pigs (*MIC2*), cattle (*BoLA-MIC1*, *2*, and *3*), humans (*MICA* and *B*), chimpanzees (*Patr-MICA/B*), rhesus macaques (*Mamu-MIC1* and *2*), and mice and rats (*Mr1*). A phylogenetic tree was constructed using the sequences corresponding to *MIC* exons 2, 3, and 4 using the neighbor joining method. The numbers on the nodes indicate the bootstrap values above 50% (n = 1000). The accession numbers of sequences are indicated in parentheses. SLA-1*0401(AF464016), one of the most common *SLA* molecules of swine, was used as an out-group. Bar below the tree indicates distance scale.

A phylogenetic tree incorporating all of the identified pig *SLA-MIC2* sequences corresponding to exons 2, 3, and 4, as well as those from humans (*MICA* and *B*), chimpanzees (*Patr-MICA/B*), rhesus macaques (*Mamu-MIC1* and *2*), cattle (*BoLA-MIC1-3*), mice and rats (*Mr1*), are shown in Fig 5. The tree depicts a low phylogenetic resolution for all sequences within a locus, particularly in the case of *SLA-MIC2*. This may suggest that the current genetic variations in *MIC* genes, including *SLA-MIC2*, have recently emerged, or that the genes have been under selective pressure to limit sequence changes. The result of phylogenetic analysis of *MIC* genes from the mammals selected for this study was consistent with reported evolutionary relationships. For example, rodent species (mice and rats) are distantly related to primates (humans, chimpanzees, and rhesus macaques) and ungulates (pigs and bovines). Interestingly, in primate branch, human *MICA* alleles are closer to *MICA/B* from chimpanzee than human *MICB*. This possibly indicates a vertical evolution of *MIC* genes in primate species. *SLA-MIC2* alleles are clustered tightly with *BoLA-MIC* genes, indicative of the close evolutionary relationship between them.

### Applications

Investigations into the diversity and evolution of *MHC* related genes is essential to understanding relationships between genetic differences, as well as the resistance and susceptibility to infectious or immune-related diseases. Bacterial infection is considered the most important cause of neonatal and post-weaning diarrhea in pigs. For example, *E*. *coli*-related diseases were ranked as the largest threat to economic loss in suckling pigs, and third largest threat to economic loss in weaned pigs [45]. In the swine industry, loss of productivity, as well as the morbidity and mortality from *E*. *coli*, costs producers enormous economic losses annually [46]. There is evidence that pathogenic strains of *E*. *coli* trigger a rapid *MICA* expression at the surface of the intestinal epithelium [47]. Understanding *SLA-MIC2* polymorphisms in pig populations and their expression may be important from the viewpoint of porcine immunogenetics. Moreover, understanding *SLA-MIC2* polymorphisms could benefit biomedical research using porcine models, considering *MIC* molecules are involved in the rejection of grafted tissues in humans [48,49].

The importance of the *MIC* genes has been highlighted by their implication in different human diseases [22,23]. Furthermore, the genetic improvement of livestock resistance to pathogens holds promise in the animal breeding arena; e.g., by the selection of pigs with an increased resistance to infectious diseases. Therefore, the typing method that we developed to analyze the *SLA-MIC2* polymorphisms, and the information that we obtained about the genetic diversity of the *SLA-MIC2* gene in pigs, could be used as novel tools, and could serve to promote other advancements in understanding porcine immune responses. Our results can also be used to establish *MIC* polymorphism for the *SLA* database in the IPD (http://www.ebi.ac.uk/ipd/mhc), which is currently only available for *HLA*.

## Supporting Information

S1 Fig The nucleotide and amino acid sequences of the *SLA-MIC2* coding region and corresponding exons. The boundaries for each exon are indicated by vertical lines.
(TIF)

S2 Fig PCR amplification results of fifteen different alleles using a set of porcine *SLA-MIC2*-specific primers. A 2512-bp segment of the genomic *SLA-MIC2* locus was amplified consistently from all alleles. The number on the top of the lane corresponds to the respective allele: 1. MIC2*01, 2. MIC2*03, 3. MIC*kn08, 4. MIC2*04, 5. MIC2*kn09, 6. MIC2*kn10, 7. MIC2*kn11, 8. MIC2*kn12, 9. MIC2*kn13, 10. MIC2*05, 11. MIC2*07, 12. MIC2*kn14, 13. MIC2*kn15, 14. MIC2*kn16, 15. MIC2*kn17, and N, negative control. The plus signs above the bands indicate the detected heterozygous PCR products.
(TIF)

S3 Fig Analysis of nucleotide polymorphisms of *SLA-MIC2* exons 2, 3, and 4 for fifteen detected alleles. Allele names are indicated on the left. Identical nucleotides are shown as a dot. The sequences were compared to a *MIC2* corresponding region (exon2: 169421–169675, exon3: 169957–170241 and exon4: 170818–171095) of a BAC sequence (accession number CT737281) from NCBI as a reference sequence. All non-synonymous mutations are outlined in grey.
(DOCX)
