# Supplementary figures and images for: Genetic Diversity and mRNA Expression of Porcine MHC Class I Chain-Related 2 (SLA-MIC2) Gene and Development of a High-Resolution Typing Method
Source: PLoS One. 2015 Aug 25;10(8):e0135922. doi: 10.1371/journal.pone.0135922 (PMC4549063; doi:10.1371/journal.pone.0135922)

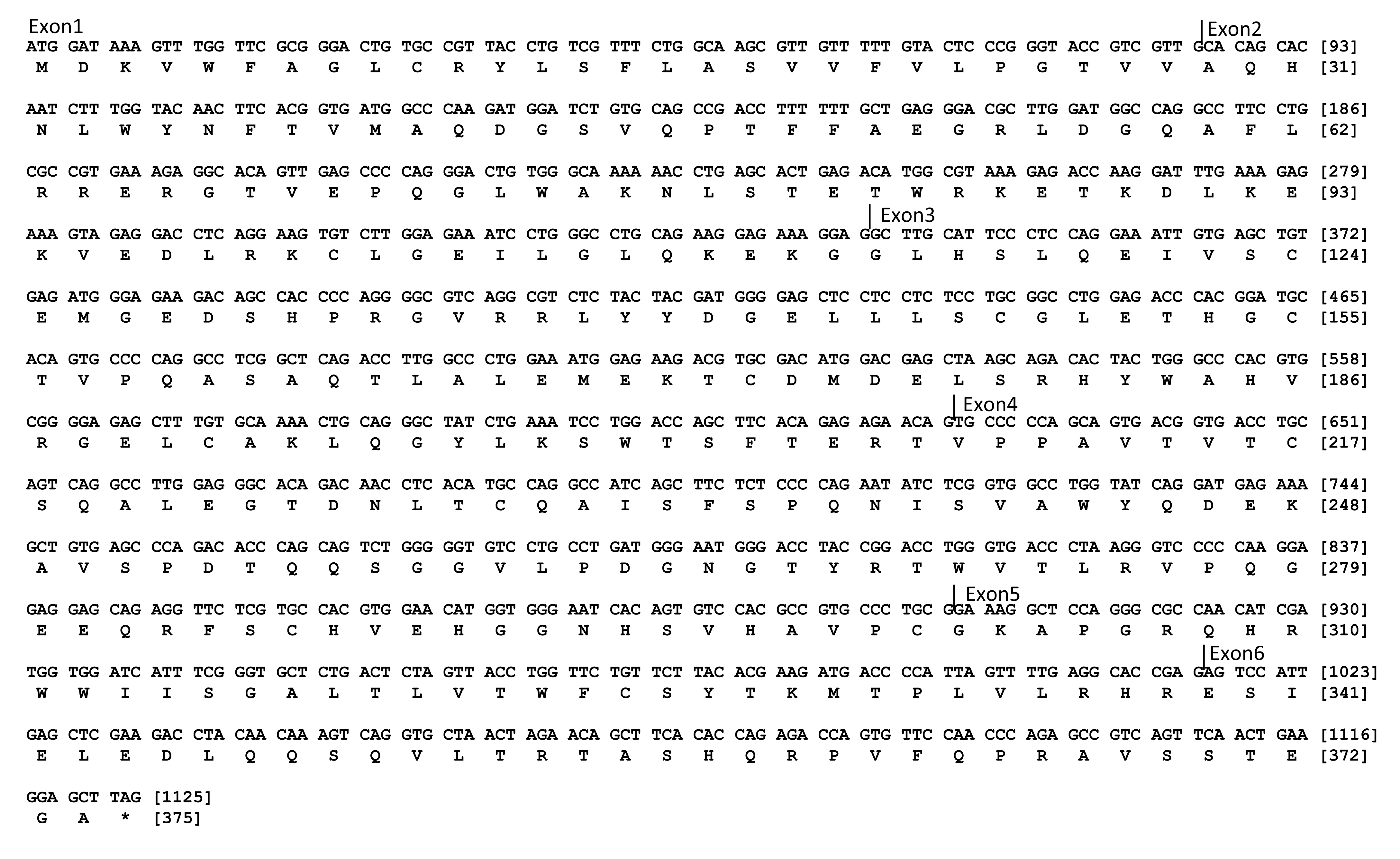

Supplement: S1 Fig — The boundaries for each exon are indicated by vertical lines. (TIF) [file pone.0135922.s001.tif]

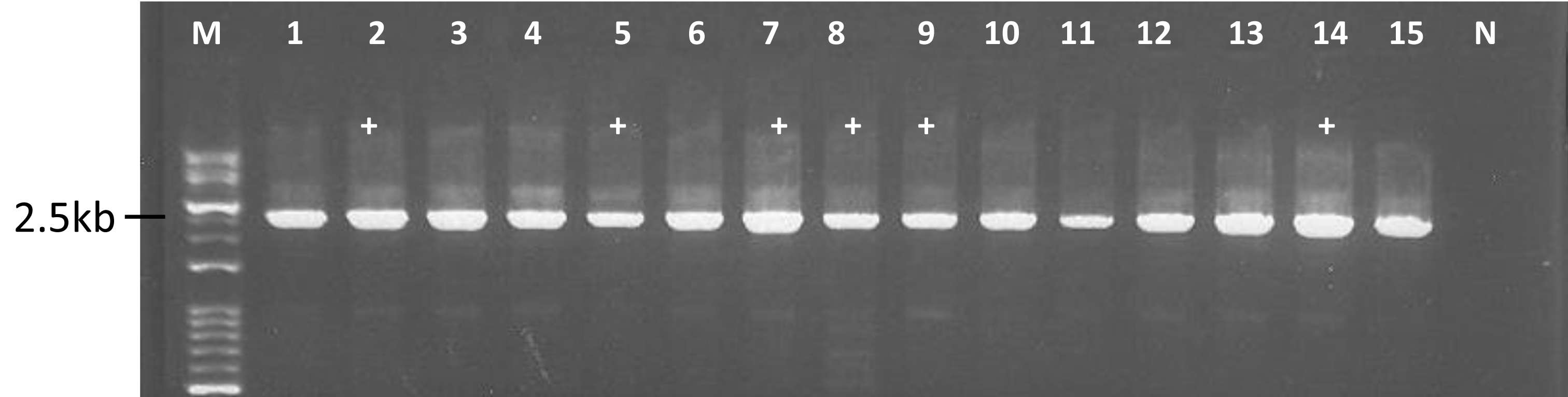

Supplement: S2 Fig — A 2512-bp segment of the genomic SLA-MIC2 locus was amplified consistently from all alleles. The number on the top of the lane corresponds to the respective allele: 1. MIC2*01, 2. MIC2*03, 3. MIC*kn08, 4. MIC2*04, 5. MIC2*kn09, 6. MIC2*kn10, 7. MIC2*kn11, 8. MIC2*kn12, 9. MIC2*kn13, 10. MIC2*05, 11. MIC2*07, 12. MIC2*kn14, 13. MIC2*kn15, 14. MIC2*kn16, 15. MIC2*kn17, and N, negative control. The plus signs above the bands indicate the detected heterozygous PCR products. (TIF) [file pone.0135922.s002.tif]
