# Supplementary material for: Genetic Diversity and mRNA Expression of Porcine MHC Class I Chain-Related 2 (SLA-MIC2) Gene and Development of a High-Resolution Typing Method
Source: PLoS One. 2015 Aug 25;10(8):e0135922. doi: 10.1371/journal.pone.0135922 (PMC4549063; doi:10.1371/journal.pone.0135922)
Supplement: S3 Fig — Allele names are indicated on the left. Identical nucleotides are shown as a dot. The sequences were compared to a MIC2 corresponding region (exon2: 169421–169675, exon3: 169957–170241 and exon4: 170818–171095) of a BAC sequence (accession number CT737281) from NCBI as a reference sequence. All non-synonymous mutations are outlined in grey. (DOCX) [file pone.0135922.s003.docx]

**Cys/Trp**

**Leu/Arg**

**Gly/Ser**

**Cys/Arg**

**Arg/Leu**

Exon 3

Exon 2

**Arg/Gln**

**Ile/Val**

Exon 4
